# Supplementary material for: Magnetic Janssen effect
Source: Nat Commun. 2021 Apr 30;12:2486. doi: 10.1038/s41467-021-22722-y (PMC8087669; doi:10.1038/s41467-021-22722-y)
Supplement: Supplementary file 1 — Supplementary Information [file 41467_2021_22722_MOESM1_ESM.pdf]

Supplemental Materials

1 Experiments

1.1 Measurement protocol

We provide here a detailed description of our mass measurements.

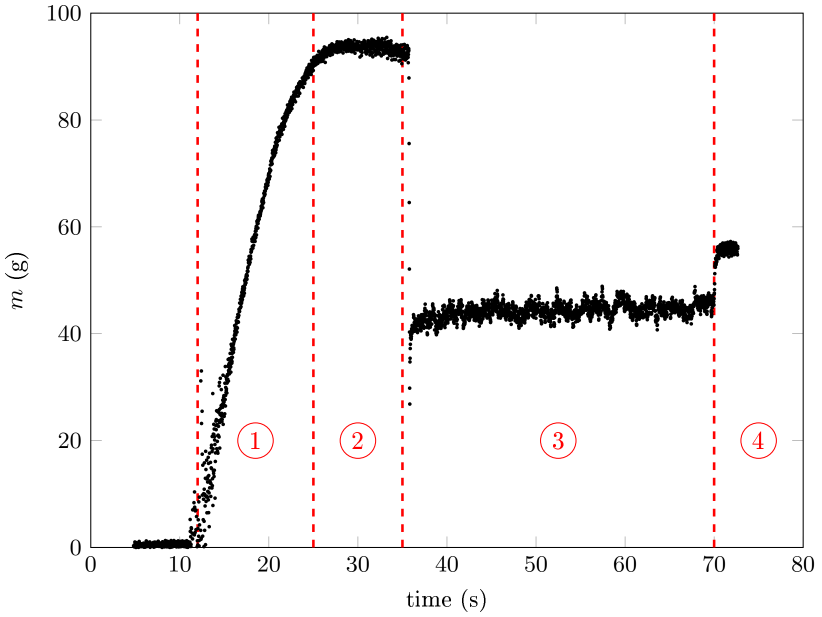

Figure S1: Typical mass measurement using the experimental setup shown in figure 1a, the mass is measured every 20 ms, and divided in several steps described in the main text.

A typical example is shown figure S1, with the following measurement protocol:

1. We fill the silo with ferromagnetic beads through a hopper fixed at the top, at a constant flow rate, with the desired mass of beads m_0 first measured using an electronic balance.

2. Once the beads are inside the silo, we remove the hopper from the top and turn on the magnetic field created by the coils in a Helmholtz configuration.

3. We then move down the vertical translation stage at a constant velocity $v = 0.2\,\mathrm{mm\,s^{-1}}$ over a distance of $\Delta h = 7\,\mathrm{mm}$. As the piston (on which the beads lay) starts to go downward, the measured mass decreases rapidly: the frictional forces along the wall are fully mobilized and the piston undergoes a decompression [2] before stabilizing at a plateau value. The actual mass of the packing m is taken as the mean value of the mass measured during the displacement and its standard deviation reflects the typical dispersion of our measurement.

4. Once the translation stage stops, the measured mass increases a bit due to the inertia of the packing slightly crushing the piston and thus the force sensor acting as a spring.

Finally, to avoid any influence of magnetic history of the beads during the experiment, the packing is demagnetized before each mass measurement, following the hysteresis protocol given in [6]. Between each measurement, the beads are placed in an 100% amplitude-modulated 20 Hz sinusoidal signal.

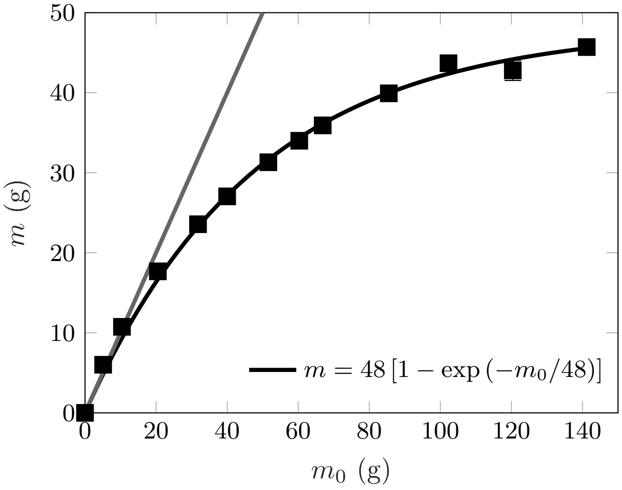

Figure S2: Apparent mass without any external magnetic field $\Psi = 0$. In this case the experimental data points are well fitted by the classical Janssen expression m_J. The measurement errors correspond to the standard deviation around the mean mass value during displacement.

1.2 Janssen Effect

We could check that without any magnetic field applied to the ferromagnetic granular column, $\Psi = 0$, its apparent mass is well described by Janssen's approach [1], predicting an exponential saturation of the measured mass: $m_J = m_\infty\,[1 - \exp(-m_0/m_\infty)]$. Fitting our experimental data by Janssen's expression of m_J, with the saturation mass m_∞ as a single free parameter, we obtain $m_\infty = 48\,\mathrm{g}$, characteristic of our granular column.

1.3 Limitations of our experimental set-up

The maximum intensity of the external magnetic field that we can reach with our set-up is $B_{\max} \approx 170\,\mathrm{G}$, leading to the dimensionless number $\Psi \approx 35$. On the other hand, the maximum height of grains, for which the applied field can be considered uniform in our set-up is of the order of $h_{\max} = 10\,\mathrm{cm}$, which corresponds to a mass around $150\,\mathrm{g}$.

1.4 Direct tunability of the apparent mass of the column

We could check that during our mass measurement when turning-off the magnetic field, the apparent mass suddenly jumped back to the value predicted by Janssen. A typical example is shown on the figure below, for a granular assembly of mass $m_0 = 40$g, with in our silo an apparent mass of $m_J = 28.2$ g without any magnetic field applied, and, when applying a magnetic field of maximum amplitude ($\Psi = 35$), an apparent mass of $m = 20.7 \pm 2.3$g. As soon as, the magnetic field is switched-off during the mass measurement, the apparent mass jumps back to the value $m = 28.4 \pm 0.9$ g, equal to m_J.

This observation on one hand confirms the robustness of our measurements and, furthermore, demonstrates our ability to tune directly the apparent mass of our ferromagnetic granular column.

2 Numerical computation of the magnetic interactions

2.1 3D random close packing generation

To generate random packings of particles, we used discrete element method simulations with the code MercuryDPM [4], where we followed the numerical filling protocol proposed by [5]. Layers of $N = 20$ randomly positioned beads are let sedimenting from a fixed distance to the top of the packing, in a tube

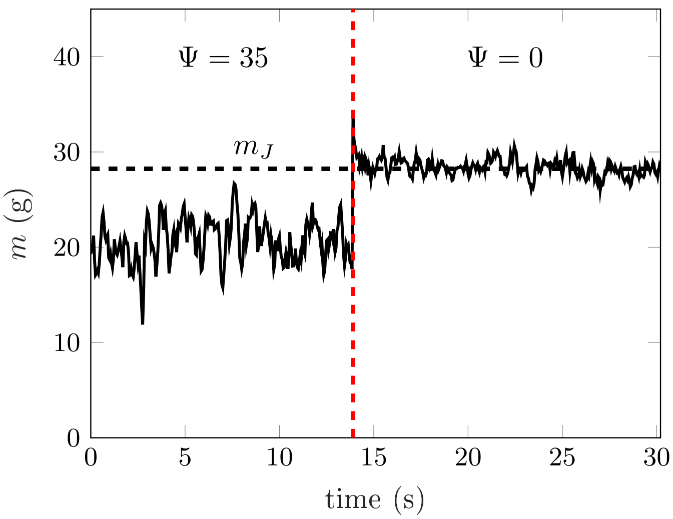

Figure S3: Switching-off the magnetic field during our mass measurement, the apparent mass of the granular columns jumps to the value predicted by Janssen' model, m_J.

geometry, until reaching the desired mass m_0. Figure S4 shows a typical example of our numerical filling protocol. The final state reached corresponds to a random close packing of filling fraction $\phi \approx 0.6$.

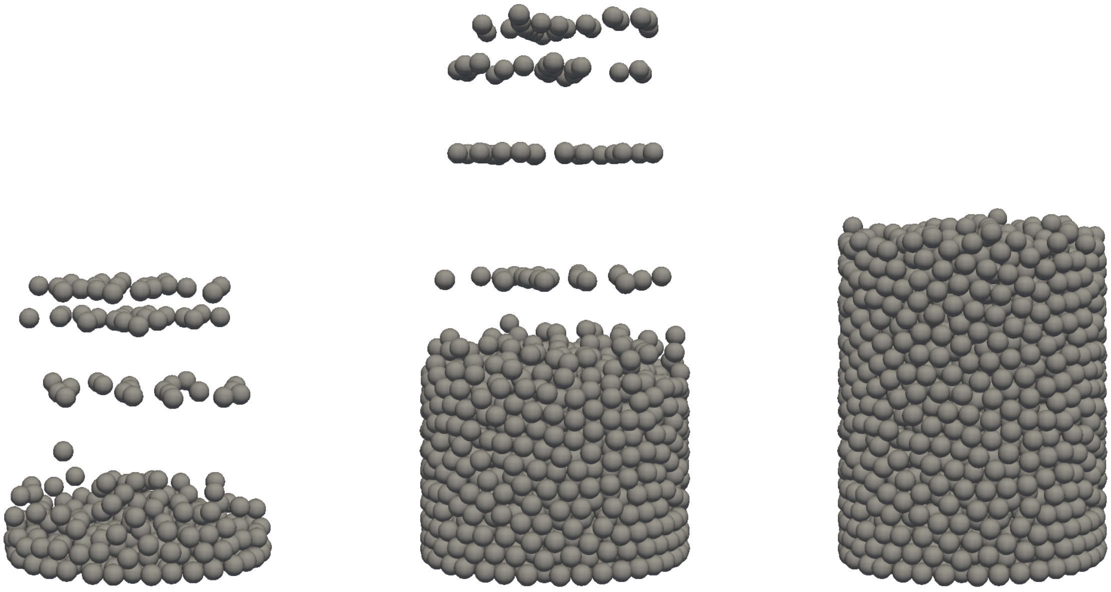

Figure S4: Filling process of the tube in our simulations, layers of 20 beads are inserted at a constant distance from the packing top until we reach the desired number of beads corresponding to a given actual mass m_0.

2.2 Computation of the magnetic pair-interactions

Once the granular packing of mass m_0 has been generated, we use the position of each particles to compute the matrix of pair-distance vectors r_{ij} and then compute all the magnetic pair interactions $\mathbf{f}_{ij}$, using the following equation (S1), where **rij** is the vector between the two beads i and j, **d** is the dipolar moment of

each bead (all equals in our case) [3]:

$$\mathbf{f}_{ij} = \frac{3\mu_0}{4\pi r_{ij}^5}\left[2\left(\mathbf{d}\cdot\mathbf{r}_{ij}\right)\mathbf{d}_j + d^2\mathbf{r}_{ij} - 5\frac{\left(\mathbf{d}\cdot\mathbf{r}_{ij}\right)^2}{r_{ij}^2}\mathbf{r}_{ij}\right], \tag{S1}$$

The force felt by each particle is then given by the sum of all pair interactions to which it is subjected.

Figure S5 provides a visual representation of those magnetic interactions within the ferromagnetic granular column, of 3 different heights, submitted to a vertical or horizontal applied magnetic field. Only a single vertical layer of ferromagnetic particles is displayed with the corresponding magnetic forces (averaged along the angular coordinate θ), they are subjected to. Red (respectively blue) arrows corresponds to positive (respectively negative) radial forces, with an intensity increasing with the norm of the force. We clearly observe that for a small packing height, those magnetic forces do not present any specific structural organization. Nevertheless, in this case of a vertical external magnetic field, those forces are mainly negative. On the other hand, for higher columns the magnetic interactions are essentially radial along the walls and cancel out within the granular packing. For this specific case of a vertical magnetic field, this radial force along the wall is clearly repulsive, *i.e.* the ferromagnetic particles at the wall push against the walls of the silo, resulting therefore in a decrease of the apparent mass of the granular column.

To go further in the analysis of the emergence of those radial forces along the walls of the silo, figure S6 shows spatial (r,z) maps of the radial component of those magnetic forces (averaged over the angular coordinate θ) inside the silo, for various packing height h, submitted to a a vertical magnetic field (figure S7 for a horizontal field), together with the corresponding evolution of the total radial force $F^r = \sum_{i,j}\mathbf{f}_{ij}\cdot\mathbf{e_r}$ with the actual mass of the packing m_o. We observe that the radial force is mainly negative for packing mass $m_0 < 30\,\mathrm{g}$, corresponding to column height $h < 2R$; then, when increasing the height of the granular packing, a vertical layer of positive radial force emerges along the wall and develops with the packing height; while the radial forces have a tendency to average to zero inside the packing.

Finally, figure S8 shows the radial force along the vertical direction z for different heights of the packing. One can observe that for high enough granular column, the radial force emerging at the walls is a constant, independent of the packing height, leading to a linear evolution of the global radial force F^r with the packing mass m_o. Some edge effects appear at the top and bottom of the silo, leading to a strong decrease of this constant radial force along z, typically over a range corresponding to a radius of the container.

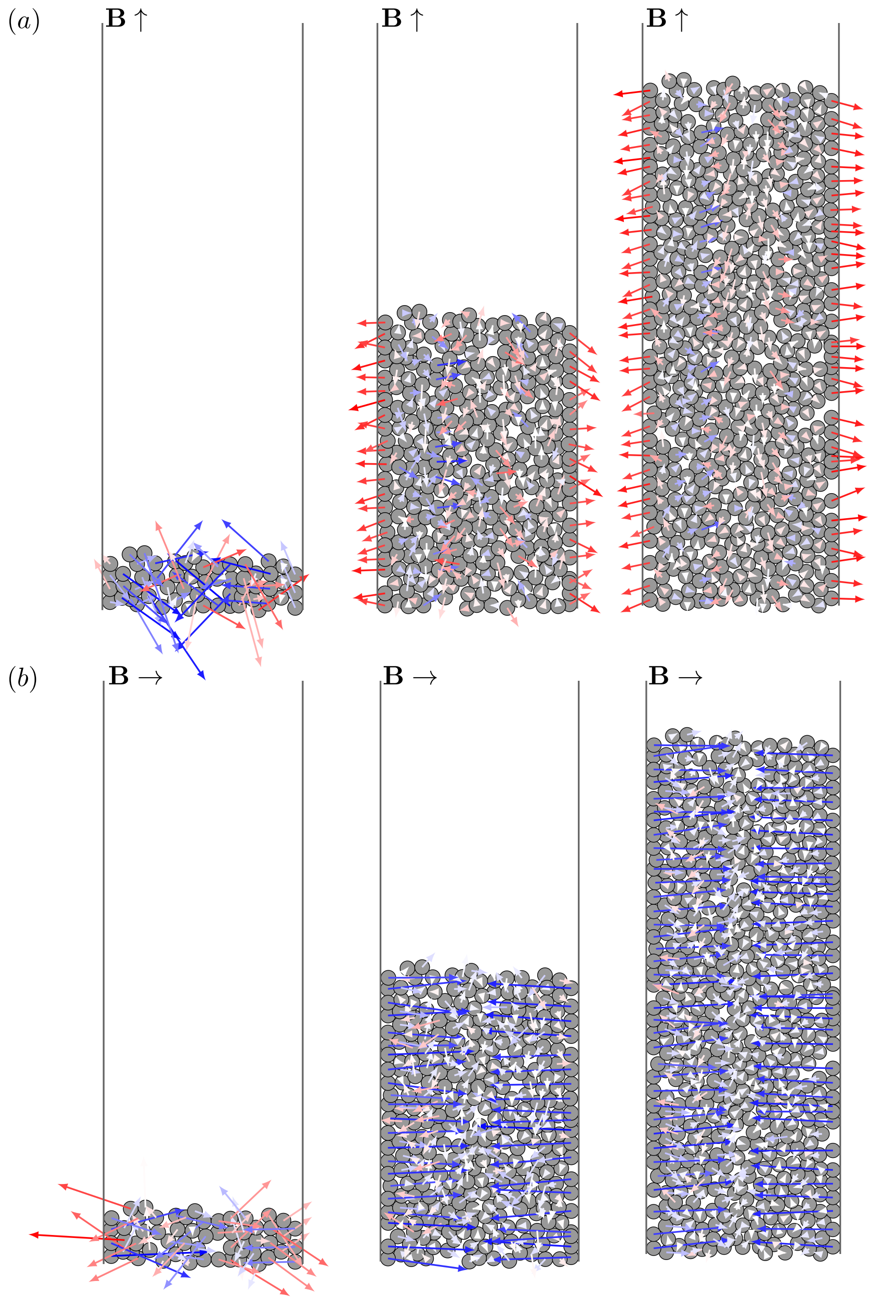

Figure S5: Visual representation of the forces exerted on each beads inside a packing layer where red (respectively blue) arrows corresponds to positive (respectively negative) radial forces, with an intensity increasing with the norm of the force. The forces have been averaged in the θ direction; the applied magnetic field is (a) vertical (b) horizontal.

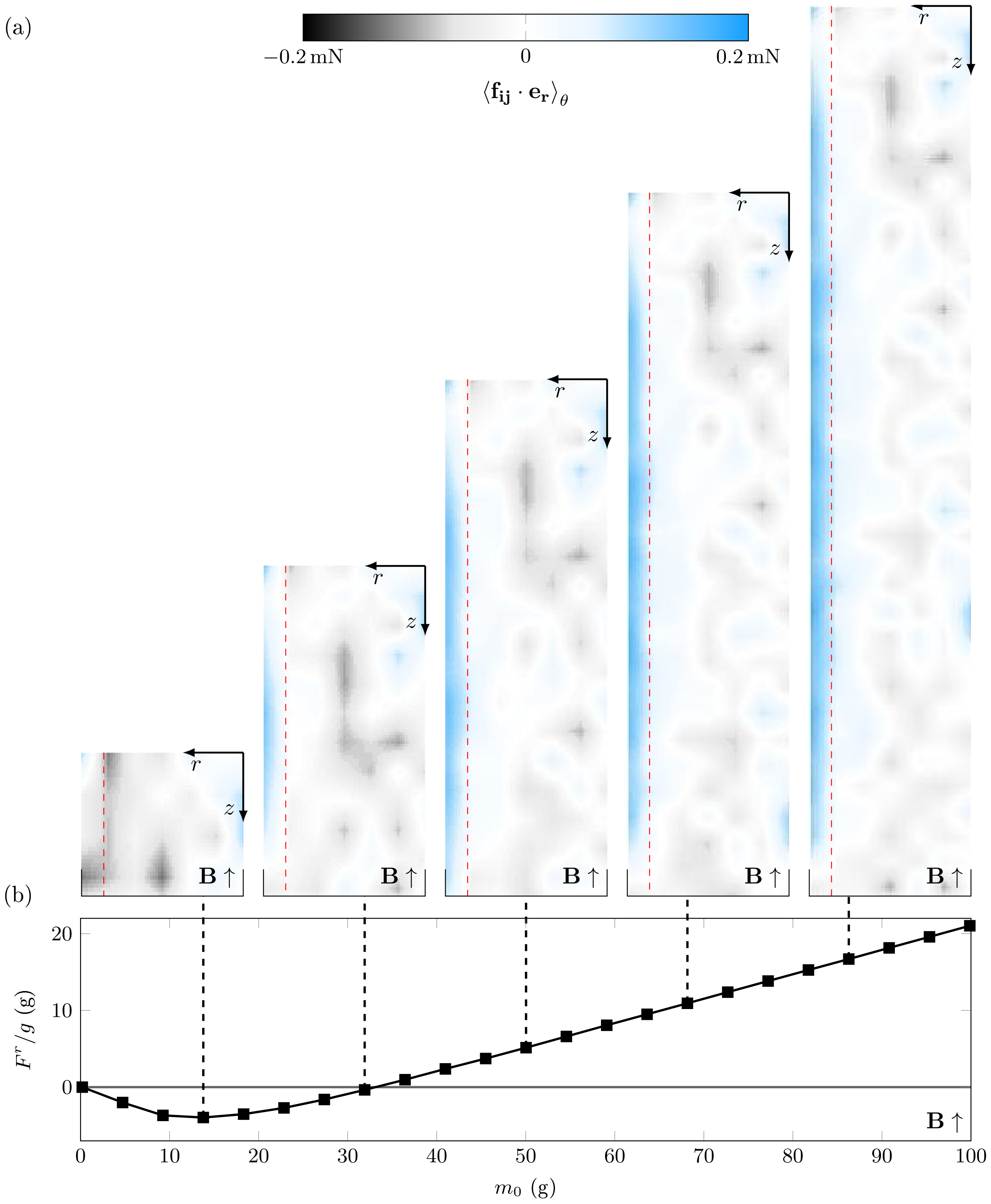

Figure S6: (a) Maps of the radial force at different packing height (or mass), averaged in the θ direction for a vertical magnetic field. A strong blue region corresponds to a positive radial force (*i.e.* pressing on the wall of the container) is observed mainly located along the boundary of the silo at $r = R$, for high enough columns $h > 2R$. For smaller ones, the radial force is mainly negative and distributed in the core of the granular packing. (b) Global radial force evolution with the packing height.

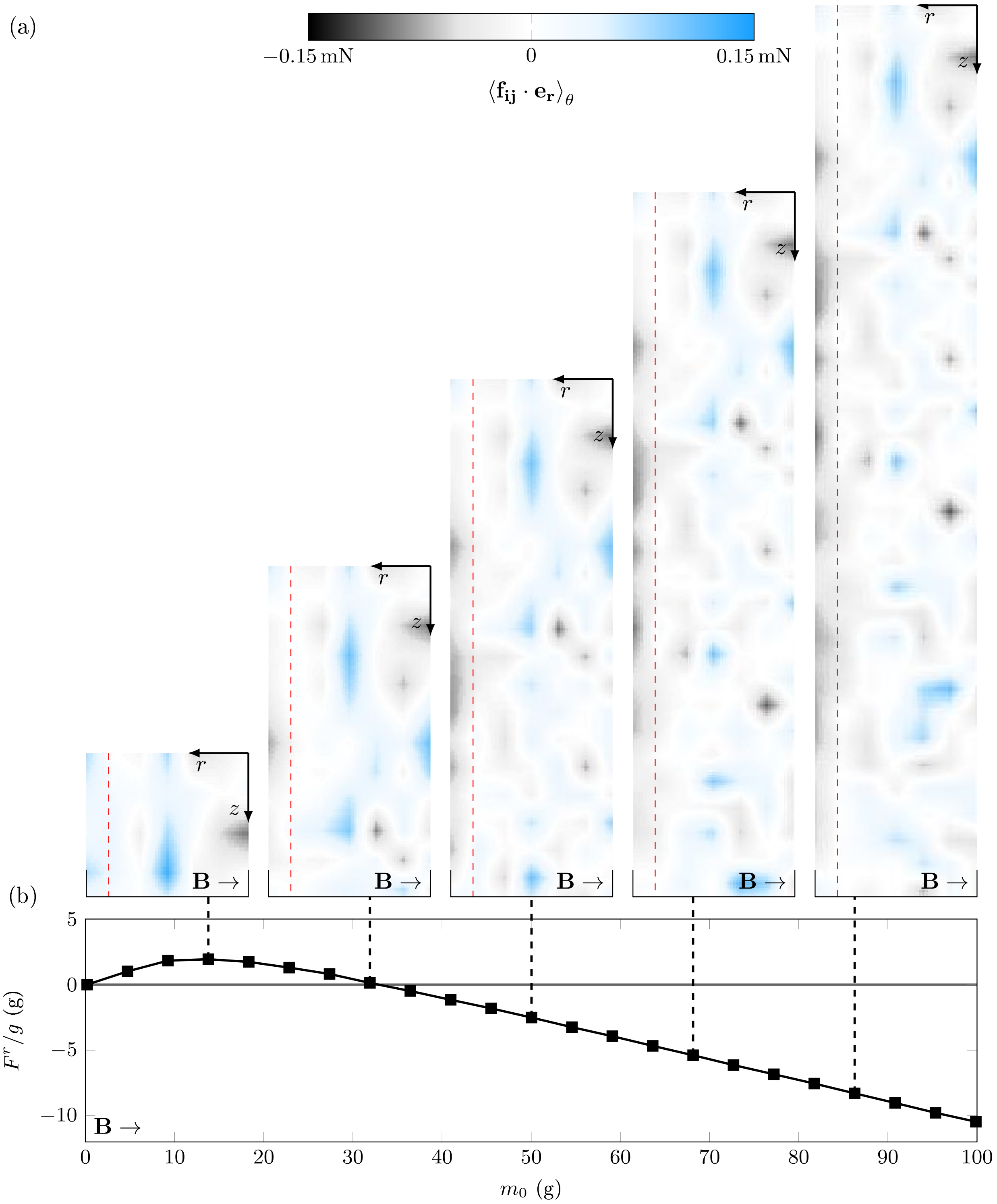

Figure S7: (a) Maps of the radial force at different packing height (or mass), averaged in the θ direction for a horizontal magnetic field. A strong blue region corresponds to a positive radial force (*i.e.* pressing on the wall of the container) is observed mainly located along the boundary of the silo at $r = R$, for high enough columns $h > 2R$. For smaller ones, the radial force is mainly negative and distributed in the core of the granular packing. (b) Global radial force evolution with the packing height.

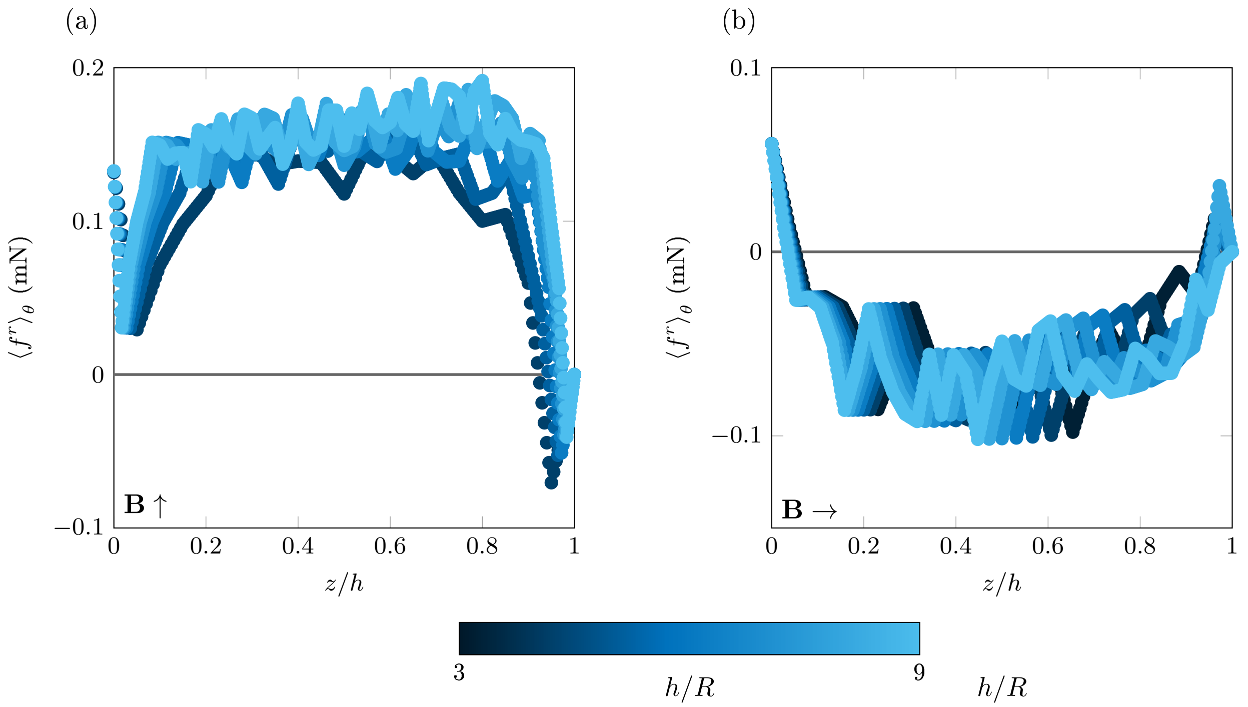

Figure S8: Vertical dependence of the radial force at $r = R$, averaged over θ, for different tube height, the force is constant apart from the top and bottom part $z < R$, $z > h - R$, where edges effects are predominant. (a) Vertical magnetic field (b) horizontal magnetic field.

References

[1] Janssen, H. A. Investigations of pressure of grain in silo. *Vereins Eutscher Ingenieure Zeitschrift* 1045–1049 (1895).

[2] Vanel, L. & Clément, E. Pressure screening and fluctuations at the bottom of a granular column. *The European Physical Journal B* **11**, 525–533 (1999).

[3] Edwards, B. F. & Edwards, J. M. Dynamical interactions between two uniformly magnetized spheres. *European Journal of Physics* **38**, 015205 (2016).

[4] Weinhart, T. et al. Fast, flexible particle simulations — an introduction to MercuryDPM. *Computer Physics Communications* **249**, 107129 (2020).

[5] Landry, J. W., Grest, G. S., Silbert, L. E. & Plimpton, S. J. Confined granular packings: Structure, stress, and forces. *Physical Review E* **67** (2003).

[6] Spaldin, N. A. Magnetic Materials (Cambridge University Press, 2009).
